# Optimized combination of circulating biomarkers as predictors of prognosis in AECOPD patients complicated with Heart Failure

**DOI:** 10.7150/ijms.52405

**Published:** 2021-02-04

**Authors:** Can Yao, Lingwei Wang, Fei Shi, Rongchang Chen, Binbin Li, Wenwen Liu, Mengjie Feng, Sinian Li

**Affiliations:** 1 Key Laboratory of Shenzhen Respiratory Diseases, Institute of Shenzhen Respiratory Diseases, Shenzhen People's Hospital (The First Affiliated Hospital of Southern University of Science and Technology, The Second Clinical Medical College of Jinan University), Shenzhen, Guangdong, China.; 2 Key Laboratory of Shenzhen Respiratory Diseases, Institute of Shenzhen Respiratory Diseases, Emergency Department, Shenzhen People's Hospital (The First Affiliated Hospital of Southern University of Science and Technology, The Second Clinical Medical College of Jinan University), Shenzhen, Guangdong, China.

**Keywords:** acute exacerbation of chronic obstructive pulmonary disease, heart failure, C-reaction protein/albumin ratio, neutrophil-lymphocyte ratio, platelet-lymphocyte ratio, prognosis

## Abstract

**Background:** Systematic inflammation, nutritional status, and cardiovascular function have been associated with the outcomes of acute exacerbation of chronic obstructive pulmonary disease (AECOPD) patients with heart failure (HF). However, the value of their relevant biomarkers in predicting mortality has not been well defined yet. We aimed to investigate the prognostic value of circulating biomarkers including C-reaction protein (CRP)/albumin (ALB), neutrophil-lymphocyte ratio (NLR), platelet-lymphocyte ratio (PLR), and N-terminal pro-brain natriuretic peptide (NT-proBNP) for AECOPD patients with HF.

**Methods:** A retrospective study was carried out in the Second Clinical College of Jinan University from January 1, 2013 to January 31, 2019. A total of 146 cases of AECOPD complicated with HF were enrolled and classified into survivor group (n=94) and non-survivor group (n=52). The baseline characteristics, CRP/ALB ratio, NLR, PLR, serum levels of NT-proBNP, and other indicators were collected. The predictors for prognosis were analyzed by multivariate logistic regression, and the ability to predict 28-day mortality was evaluated by receiver operating characteristics curve (ROC) and the area under the curve (AUC).

**Results:** The patients in non-survivors had significantly higher levels of CRP, CRP/ALB, NLR, PCT and NT-proBNP, but lower ALB levels compared to the survivors [111.7 (56.9, 186.5) VS. 43.8 (10.3, 96.1) mg/L, 4.6 (2.0, 8.0) VS. 1.4 (0.3, 3.4), 22.2 (11.1, 40.1) VS. 12.0 (6.2, 24.8), 2.6 (0.2, 10.3) VS. 0.08 (0.1, 0.5) ng/ml, 17912.5 (9344.0, 34344.5) VS. 9809.0 (4415.9, 16387.2) ng/ml, 25.8 (23.2, 30.5) VS. 30.7 (27.9, 34.1) g/L; *P* < 0.001, <0.001, 0.001, <0.001, <0.001, and < 0.001, respectively]. No significant difference in PLR was found between the two groups (*P*=0.413). The logistic analysis revealed that CRP/ALB (OR=1.303, 95%CI: 1.145-1.483, *P*<0.001), NT-proBNP (OR=1.041, 95%CI: 1.010-1.073, *P*=0.009) and NLR (OR=1.010, 95%CI: 0.999-1.022, *P*<0.001) are independent risk factors for predicting the 28-day mortality. The AUC of the ROC curves were 0.768, 0.767, 0.757, 0.723, 0.716, and 0.668 for CRP/ALB, PCT, CRP, NT-proBNP, ALB, and NLR, respectively. The combination of CRP/ALB, NLR and NT-proBNP as biomarkers was shown to have better accuracy for predicting prognosis (AUC=0.830, 95%CI: 0.761-0.899, *P*<0.001), with a higher specificity of 80.8% and specificity of 77.7% as compared with each single biomarkers.

**Conclusions:** High levels of NLR, CRP/ALB and NT-proBNP may be clinical usefully predictors for death in AECOPD patients with HF. Combination of NLR with CRP/ALB and NT-proBNP can provide a higher accuracy for predicting 28-day mortality in these patients.

## Introduction

Chronic Obstructive Pulmonary Disease (COPD), a common and progressive lung disease, is characterized by persistent respiratory symptoms and airflow limitation. COPD is currently a major cause of mortality and morbidity worldwide, and is estimated to become the third leading cause of death by 2020 1. Acute exacerbation of COPD (AECOPD) is the most common cause of increasing hospitalization rate, and accounts for increasing death among patients with COPD 2,3. Meanwhile, heart failure (HF) is one of the most critical comorbidities in patients with AECOPD. It was reported that the prevalence of COPD patients with systolic or diastolic HF ranges from 20 to 70%, which is associated with higher incidence of hospitalization and mortality compared to patients without HF 2,4. Therefore, early identification of patients with poor prognosis is very critical to the optimization of personalized therapies. Circulating biomarkers, which are easily accessible from peripheral blood, might provide important information for clinical decision, but their roles in predicting prognosis in AECOPD complicated with HF has not been well be defined yet.

It has been recognized that systematic inflammation, nutritional status, and cardiovascular function play essential roles in the development of AECOPD 5-7. N-terminal pro-brain natriuretic peptide (NT-proBNP) is a well-established biomarker in determining diagnosis and prognosis in HF 6,7, and has also been found to be increased in AECOPD with or without HF 8,9. In addition, several novel inflammatory markers, such as C-reaction protein (CRP)/albumin (ALB) ratio, neutrophil-lymphocyte ratio (NLR), and platelet-lymphocyte ratio (PLR) have also been recently used for monitoring of inflammatory conditions. CRP is one of the non-specific proteins that represent systemic inflammation, while a low ALB level may reflect a temporary condition that requires medical intervention. It has been shown that CRP/ALB ratio can be used as a long-term marker of prognosis in septic patients, and provides more stable information than CRP or ALB alone 10. Moreover, both NLR and PLR are proved to play an important role in outcome prediction and prognosis of COPD patients and HF patients, respectively 3,6-9. However, the prognostic value of these indicators in AECOPD patients with HF are still uncertain. This study aimed to demonstrate the utility of circulating biomarkers including CRP/ALB ratio, NLR, PLR, and NT-proBNP in predicting prognosis in AECOPD patients with HF.

## Methods

### Patients and study design

A total of 146 patients hospitalized for AECOPD complicated with HF was consecutively collected from January 1, 2013 to January 31, 2019. Demographic and medical data were retrospectively obtained from electronic patient records and paper notes of Shenzhen Institute of Respiratory Diseases, the Second Clinical College of Jinan University in Shenzhen, China. The study protocol (LL-KY-201932) was approved by the Ethics Committee of the Second Clinical College of Jinan University. Requirement for patient consent was waived.

Patients with COPD were identified by International Classification of Diseases, Tenth Revision (ICD-10) principal diagnosis codes J44.900; exacerbations were defined as cases' hospitalized with the codeJ44.100 and J44.101 as a main reason for admission; HF was identified by the codeI50.000, I50.001, I50.002, I50.100, I50.101, I50.102, I50.103, I50.907, and I50.900. We obtained data regarding baseline demographics, staging of COPD according to Global Initiative for Chronic Obstructive Lung Disease (GOLD), laboratory parameters, chest radiographs, and echocardiograms at the time of admission.

**Figure 1:**
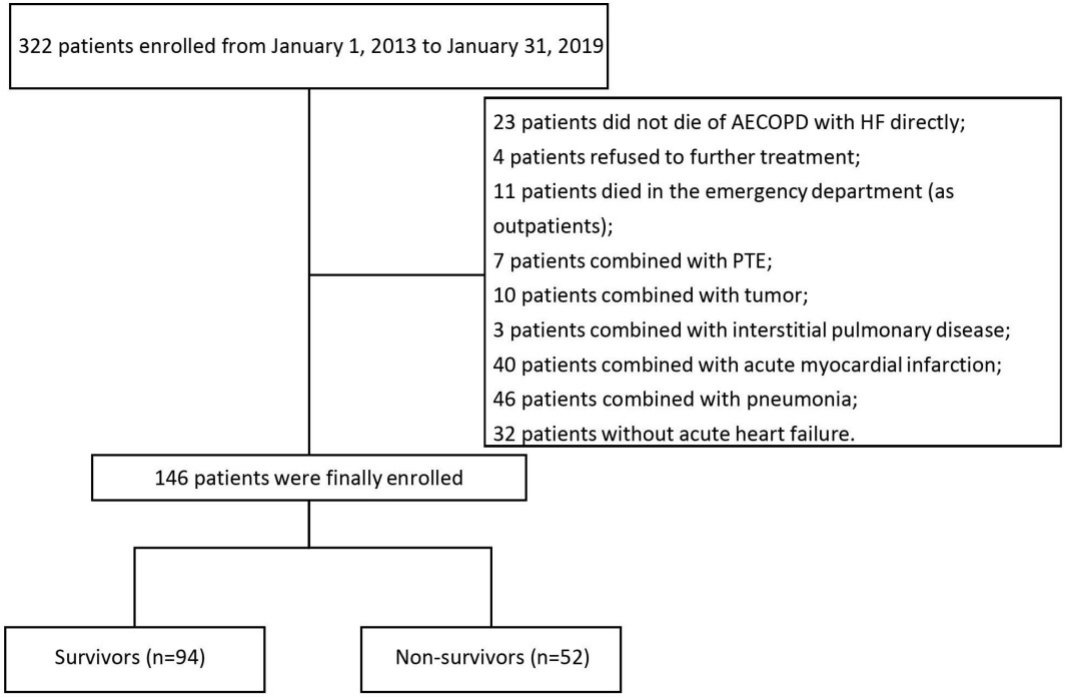
Screening and enrollment of the study.

The inclusion criteria was as follows: 1) diagnosis of AECOPD, defined as an acute worsening of respiratory symptoms that lead to need additional therapy 1; 2) HF occurred during the hospitalization and the diagnosis of HF was in accordance with the 2016 ESC Guidelines 11. We excluded patients with any of the following characteristics: asthma, pneumonia, pulmonary thromboembolism (PTE), tuberculosis, acute respiratory distress syndrome, rheumatic diseases, cancer, valvular heart disease, and acute myocardial infarction. We also excluded patients who died in the emergency room because the measurements were not available for these patients. More details were shown in the Figure 1.

### Data collection

We collected clinical data and laboratory indexes of all subjects including age, sex, smoking status, comorbidities, left ventricular ejection fraction (LVEF), history of exacerbation, GOLD grade, COPD assessment test (CAT) score, length of stay (LOS) in hospital, LOS in ICU, probability of endotracheal intubation, the initial value of ALB and the New York Heart Association (NYHA) functional classification. Serum CRP levels, PCT levels, NT-proBNP levels and routine blood test results including neutrophils, lymphocytes and platelets were obtained within 2 hours of admission to the hospital.

### Outcomes

Our primary outcome is 28-day mortality. It was assessed both from the medical records and phone calls. All the adverse events were adjudicated by clinicians. All the patients were divided into survivors group (n=94) and non-survivors group (n=52) according to their survival conditions.

### Statistical analysis

Statistical Package for Social Sciences (SPSS), version 13.0 (SPSS Inc, Chicago, IL, USA) was used for statistical analysis. Measurement data from normal distribution was expressed as the mean ± standard deviation and independent-samples *t* test was used to assess significant differences. Non-normally distributed continuous data was expressed as the median (interquartile range, IQR), and two-sample Wilcoxon Rank Sum test was used to analyze. Descriptive data was also expressed as the median (IQR). Chi-square test or Fisher's exact test was for categorical variables. Multivariate logistic regression analysis was used to check the independent correlation factors. Receiver operating characteristic (ROC) curves were generated to evaluate the biomarkers for predicting the mortality hazard. The Youden index was used to determine the optimal cutoffs of variables including CRP/ALB, NLR, and NT-proBNP. The combination tests based on logistic models were performed to assess the prognostic performance of biomarker combinations. A *P* value of less than 0.05 (*P*< 0.05) was considered as a statistically significant difference.

## Results

### Baseline characteristics

A total of 146 AECOPD patients complicated with HF were included in this analysis. The 28-day mortality in our study is 35.6%. The number of all patients with NYHA class II, III or IV were 29, 71 and 46, and GOLD grade 2, 3, or 4 were 41, 85 and 20, respectively. The baseline characteristics of the survivors and non-survivors are shown in Table 1. The mean age of the study patients was 79±10 years, 75% were male. However, there was no significant difference in age, gender, smoking status, complications, GOLD grade, baseline CAT score, LVEF and median hospital LOS, between the two groups. Among the 52 non-survivors, 38 (73%) needed for endotracheal intubation. Non-survivors were more likely to be in NYHA III - IV (Non-survivors VS. Survivors: 96.2% VS. 71.3%, *P* < 0.001), have a longer median ICU LOS (Non-survivors VS. Survivors: 5 VS. 1 day, *P* < 0.001), and have a higher probability of endotracheal intubation (Non-survivors VS. Survivors: 73% VS. 18%, *P* < 0.001).

### Biomarker levels and outcomes

The comparison of laboratory indexes between the two groups was shown in Table 2. The patients who survived in hospital had lower levels of serum CRP [survivors VS. non-survivors: 43.8 (10.3, 96.1) VS. 111.7 (56.9, 186.5) mg/L,*P* < 0.001], CRP/ALB [survivors VS. non-survivors: 1.4 (0.3, 3.4) VS. 4.6 (2.0, 8.0), *P* < 0.001], NLR [survivors VS. non-survivors: 12.0 (6.2, 24.8) VS. 22.2 (11.1, 40.1), *P* = 0.001], serum PCT [survivors VS. non-survivors: 0.08 (0.1, 0.5) VS. 2.6 (0.2, 10.3) ng/ml, *P* < 0.001], and serum NT-proBNP [survivors VS. non-survivors: 9809.0 (4415.9, 16387.2) VS. 17912.5 (9344.0, 34344.5) ng/ml, *P* < 0.001]. There were no differences in PLR between survivors and non-survivors [survivors VS. non-survivors: 257.3 (160.3, 416.8) VS. 270.4 (161.9, 408.1), *P* = 0.474]. We also noted that the patients who survived had higher ALB levels than the patients who died [30.7 (27.9, 34.1) VS. 25.8 (23.2, 30.5) g/L, *P* < 0.001]. The multivariate logistic analysis suggested that CRP/ALB (OR = 1.303; 95% CI: 1.145-1.483; *P* = 0.001), NT-proBNP (OR = 1.041; 95% CI: 1.010-1.073; *P* = 0.009) and NLR (OR = 1.010; 95% CI: 0.999-1.022; *P* = 0.001) can be independent risk factors for predicting the mortality of included subjects. Prognostic model equation was LogitP=-2.579+0.265CRP/ALB+0.041NT-proBNP+0.010NLR. More details in both groups were shown in Tables 2 and 3.

### Predictive ability of biomarkers

**Figure 2:**
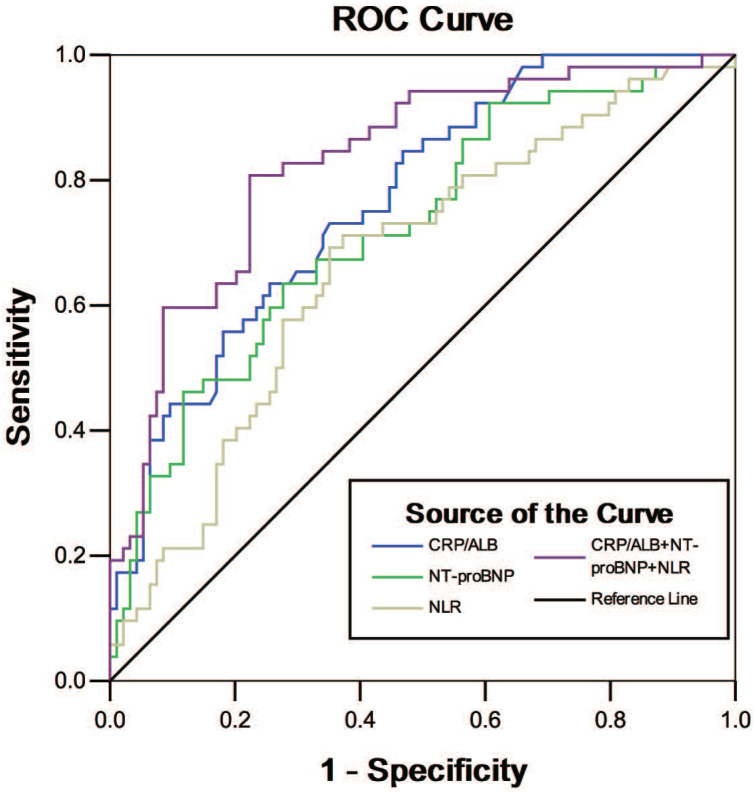
ROC curves of the circulating biomarkers for predicting 28-day mortality of acute exacerbation of chronic obstructive pulmonary disease (AECOPD) patients with heart failure (HF). The receiver operating characteristic (ROC) curves for single biomarkers and their combinations had the following areas: C-reactive protein (CRP)/albumin (ALB) ratio, 0.768; neutrophil-lymphocyte ratio (NLR), 0.668;N-terminal pro-brain natriuretic peptide (NT-proBNP), 0.723; and combination of CRP/ALB, NLR and NT-proBNP, 0.830.

To investigate the predictive abilities of biomarkers, ROC curve analysis was constructed (Table 4 and Figure 2). The AUC of CRP/ALB (AUC 0.768, 95% CI 0.684-0.850, *P* < 0.001) was much greater than other single biomarkers. The optimal cutoff value for CRP/ALB was 2.48, with sensitivity of 73.1%, and specificity of 64.9%; 16.83 for NLR with 69.2% sensitivity and 64.9% specificity (AUC 0.668, 95% CI 0.577-0.759, *P* = 0.001); 0.59 for PCT with 67.3% sensitivity and 80.9% specificity (AUC 0.767, 95% CI 0.684-0.850, *P*< 0.001); and 14265.25 for NT-proBNP with 63.5% sensitivity and 72.3% specificity (AUC 0.723, 95% CI 0.637-0.809, *P*< 0.001). We further investigated the prognostic value of different biomarker combinations: combining CRP/ALB with NT-proBNP increased predictive sensitivity (75.0%) and specificity (83.0%), with AUC of 0.821 (95% CI 0.751-0.892, *P* < 0.001). The predictive value of any two biomarker combinations was accurate with a sensitivity less than 80%. Notably, ROC curve analysis revealed, that the prediction of 28-day mortality was improved markedly by the combination of CRP/ALB, NLR, and NT-proBNP (sensitivity 80.8%, specificity 77.7%), with the best AUC of 0.830 obtained (95% CI 0.761-0.899, *P* < 0.001). The predictive accuracy of other single marker or a marker combination is listed in Table 4.

According to the ROC curve analysis, we evaluated the mortality in AECOPD patients with HF above and below the optimal cut-off of NT-proBNP. We found that more than 50% patients died when NT-proBNP above 14265.25 ng/ml (55.9%) and most of patients survived when NT-proBNP below 14265.25 ng/ml (78.2%). More details were shown in Table 5.

## Discussion

In this study, we showed the better performance of combined circulating biomarkers in predicting mortality in AECOPD patients with HF. To our knowledge and by searching Pubmed, this study is the first report on using multiple blood-based biomarkers related to systematic inflammation, nutritional status, and cardiovascular function to predict mortality in AECOPD patients with HF. Our results revealed that AECOPD patients with HF who died had significantly higher levels of CRP/ALB, NLR, NT-proBNP, and higher NYHA functional classification compared with patients who survived. Furthermore, the combination of CRP/ALB, NLR, and NT-proBNP could potentially be used to predict 28-day mortality in AECOPD patients with HF.

Our study showed that the 28-day mortality in AECOPD patients with HF was 35.6%. According to a previous study, the mortality in COPD patients with HF was significantly higher than those who without HF (25.6% vs. 12.1%) and HF can be a strong independent predictor of all-cause mortality in patients with COPD 12. Another study showed that estimated eight years mortality in patients with COPD coexisting heart failure was seven times higher than in patients with COPD alone with an odds ratio of 7.06 (95% CI 3.88-12.84) 13. Therefore, it was suggested that HF has an important impact on mortality in patients with COPD. HF should be recognized early and treated properly which may improve survival in COPD patients combined with HF.

Previous studies have shown that CRP could be used as an independent influencing factor to evaluate the control level of COPD chronic inflammation in patients 14-16, while ALB is a prognostic marker of infection-related disease 17. Our study observed that high CRP and PCT levels as well as low ALB level were associated with death in AECOPD patients with HF. This data confirmed that non-survivors have more severe systematic inflammatory response and lower nutrient intake than survivors. Although CRP and ALB have good prognostic significance for AECOPD patients, the early inflammatory response and nutritional status of the body may not change in equal proportion. Therefore, CRP/ALB ratio has been recently identified as a valuable predictor of prognosis in patients with various diseases such as tumor and sepsis 10,17,18, whereas its prognostic role remains unclear in AECOPD patients with HF. In our study, we showed that CRP/ALB ratio was significantly higher in non-survivors than in survivors (*P*< 0.001). The sensitivity and specificity of CRP/ALB ratio were better than other biomarkers in predicting 28-day mortality with a cut‐off value of 2.48. It showed that low-grade systemic inflammation and poor nutrition may play an essential role in leading to aggravation of disease. Furthermore, CRP/ALB ratio was proved as an independent risk predictor for the prognosis of AECOPD patients with HF in this study.

NT-proBNP is an important biomarker in the diagnosis of HF, and represents a novel indicator that identifies the risks of COPD exacerbation. In our study, we observed NT-proBNP level and NYHA functional classification were significantly higher in the non-survivors. Though, LVEF was similar between the two groups. Our finding also suggested that NT-proBNP is an independent death risk factor in AECOPD patients with HF. This result may be in favor of the results of previous studies 19,20. Moreover, our study demonstrated that NT-proBNP level is significantly associated with 28-day mortality, but its relevance to mortality is inferior to that of CRP/ALB. Above the cut-off of 14265.25 ng/ml, a higher mortality was observed in AECOPD patients with HF. Therefore, increasing NT-proBNP may indicate a poor prognosis for patients. It suggested that dynamic detection of NT-proBNP levels may be helpful in predicting the prognosis of AECOPD patients with HF, while the cardiovascular functions cannot better assess patients' mortality as compared with other factors such as systematic inflammation and nutrition.

We found that elevated NLR is also a strong predictor of poor outcomes in AECOPD patients with HF. In consistent with our findings, the range of NLR levels have been determined in COPD patients with different conditions (eg. acute respiratory failure, chronic respiratory failure, and existence of pneumonia) in previous studies. Furthermore, our study suggests higher NLR is related to 28-day mortality in AECOPD patients with HF. It is worth noting that the cut-off value of 16.83 for NLR was nearly two times higher in the AECOPD subjects with HF than in the COPD subjects in Yao et al's study 3. NLR is valuable in assessing the severity of COPD attack and heart function, and this may help to indicate the outcomes of AECOPD complicated with HF. Even though the predictive ability of NLR and the AUC was less than the other inflammatory biomarkers including CRP/ALB and PCT, the combination of NLR and CRP/ALB with NT-proBNP increased prognostic accuracy for 28-day mortality in AECOPD patients with HF. It was suggested that NLR level should be explained with other inflammatory biomarker such as CRP/ALB and heart failure indicator to improve the ability of predicting mortality risk.

PLR is another indicator for systemic inflammatory disorders 21. El-Gazzar et al 19 found that elevated PLR together with NLR were significantly associated with smoking index, COPD stage and dyspnea severity in stable COPD patients. However, conflicting with these findings, our study did not find the statistically significant difference in PLR between the survivors and non-survivors. The role of PLR in AECOPD complicated with HF and the reason why no difference of PLR ratios was found between the two groups is not clear.

However, there were some limitations in our study. The small sample size and the limited number of events were major limitations, and the selection of patients was based only on the ICD codes which were related to selection bias. Moreover, we only collected the circulating biomarkers once at baseline, which cannot represent the underlying changes of systematic inflammation, nutritional status, and cardiac function in patients. Because this is a retrospective study, some relevant information such as exercise tolerance, modified Medical Research Council dyspnea scale (mMRC), and pulmonary function test were not available. Nevertheless, the strength of our study is that we assessed the predictive value of multiple mortality-associated biomarkers, taking inflammation, nutrition and cardiovascular function into account, some of which were left out of the previous studies.

## Conclusions

In summary, we suggest that the levels of NLR, CRP/ALB, and NT-proBNP may be used to be prognostic markers for assessing the severity of disease and predicting 28-day mortality in AECOPD patients with HF. The combination of NLR, CRP/ALB, and NT-proBNP may significantly increase the mortality prediction, and provide prognostic information for future studies of different population of AECOPD patients.

## Figures and Tables

**Table 1:** Clinical summary of the AECOPD patients with HF

Clinical characteristic	Overall (n=146)	Non-survivors (n=52)	Survivors (n=94)	*χ*^2^/*t*/*Z*	*P*-value
Age, year, mean±SD	79±10	81±12	78±8	1.680	0.096
**Gender, n (%)**					
Male	109 (75)	42 (81)	67 (73)	1.595	0.207
Female	37 (25)	10 (19)	27 (27)		
**Smoking status, n (%)**					
Current/ever	81 (56)	27 (52)	54 (57)	0.414	0.520
Never	65 (44)	25 (46)	40 (43)		
**With hypertension or diabetes, n (%)**					
Yes	107 (73)	39 (75)	68 (72)	0.121	0.728
Not	39 (27)	13 (25)	26 (28)		
Baseline CAT score, mean±SD	23±5	24±5	23±4	1.752	0.082
GOLD grade, 2/3/4, n (%)/n (%)/n (%)	41 (28.1)/85 (58.2)/20 (13.7)	13 (25.0)/33 (63.5)/6 (11.5)	28 (29.8)/52 (55.3)/14 (14.9)	0.930	0.628
NYHA classification, II/III/IV, n (%)/n (%)/n (%)	29 (19.9)/71 (48.6)/46 (31.5)	2 (3.8)/25 (48.1)/25 (48.1)	27 (28.7)/46 (48.9)/21 (22.3)	4.090	0.000*
**HF classification, n (%)**					
Systolic heart failure	88 (60.3)	35 (67.3)	53 (56.4)	2.637	0.268
Diastolic heart failure	28 (19.2)	10 (19.2)	18 (19.1)		
Both	30 (20.5)	7 (13.5)	23 (24.5)		
LVEF (%), mean±SD	49.64±11.90	48.65±11.68	51.79±12.34	1.070	0.288
Hospital LOS, day, median (IQR)	11 (6, 21.25)	16 (5, 23)	10.5(7, 19)	0.254	0.800
ICU LOS, day, median (IQR)	2 (2, 9)	5 (1, 16.5)	1 (0, 5)	4.062	0.000*
**Endotracheal intubation, n (%)**					
Yes	55 (38)	38 (73)	17 (18)	43.120	0.000*
Not	91 (62)	14 (27)	77 (82)		

Note: Data presented as mean ± SD and median (interquartile range). Abbreviations: AECOPD, acute exacerbation of chronic obstructive pulmonary disease; HF, heart failure; SD, Standard Deviation; NYHA classification, New York Heart Association classification; CAT, COPD assessment test; LVEF, left ventricular ejection fraction; LOS, length of stay; ICU, Intensive Care Unit; IQR, interquartile range. *: *P < .*05.

**Table 2:** Comparison of Laboratory indexes between the study groups

Laboratory indexes	Overall (n=146)	Non-survivors (n=52)	Survivors (n=94)	*t* /*Z*	*P*-value
CRP, mg/L, mean±SD	65.8 (19.9, 150.1)	111.7 (56.9, 186.5)	43.8 (10.3, 96.1)	4.689	0.000*
ALB, g/L, mean±SD	29.7 (25.5, 33.3)	25.8 (23.2, 30.5)	30.7 (27.9, 34.1)	4.680	0.000*
CRP/ALB, mean±SD	2.3 (0.7, 5.2)	4.6 (2.0, 8.0)	1.4 (0.3, 3.4)	4.880	0.000*
NLR, median (IQR)	14.9 (7.0, 28.3)	22.2 (11.1, 40.1)	12.0 (6.2, 24.8)	3.349	0.001*
PCT, ng/ml, median (IQR)	0.23 (0.1, 2.6)	2.6 (0.2, 10.3)	0.08 (0.1, 0.5)	5.364	0.000*
NT-proBNP, ng/ml, median (IQR)	11997.3 (6128.2, 22030.7)	17912.5 (9344.0, 34344.5)	9809.0(4415.9, 16387.2)	4.446	0.000*
PLR, mean±SD	258.3 (160.8, 417.2)	270.4 (161.9, 408.1)	257.3 (160.3, 416.8)	0.718	0.474

Note: Data presented as median (interquartile range). Abbreviations: CRP, C-reaction protein; SD, Standard Deviation; ALB, Albumin; CRP/ALB, C-reaction protein/Albumin; NLR, Neutrophil-lymphocyte ratio; IQR, interquartile range; PCT, procalcitonin; NT-proBNP, N terminal pro B-type natriuretic peptide; PLR, Platelet-lymphocyte ratio. *: *P< .*05.

**Table 3:** Multivariate logistic regression analysis of AECOPD patients with HF

Indicator	OR	95%CI	*P*-value
CRP	1.003	0.989~1.017	0.697
ALB	0.987	0.890~1.094	0.800
CRP/ALB	1.303	1.145~1.483	0.000*
NLR	1.010	0.999~1.022	0.000*
PCT	0.998	0.965~1.033	0.928
NT-proBNP	1.041	1.010~1.073	0.009*
Age	0.951	0.973~1.030	0.552
CAT score	1.064	1.008~1.124	0.132
NYHA classification	1.006	0.964~1.049	0.081
MV	1.166	0.541~2.512	0.696

Abbreviations: AECOPD, acute exacerbation of chronic obstructive pulmonary disease; CRP/ALB, C-reaction protein/Albumin; NLR, Neutrophil-lymphocyte ratio; NT-proBNP, N terminal pro B-type natriuretic peptide; PCT, procalcitonin; CRP, C-reaction protein; ALB, Albumin; MV, mechanical ventilation; NYHA classification, New York Heart Association classification; CAT, COPD assessment test; OR, odds ratio; CI, Confidence interval. *: *P< .*05.

**Table 4:** Prognostic value of the biomarkers

Indicator	AUC	95% CI	Cut-off	Sensitivity	Specificity	*P*-value
CRP (mg/L)	0.757	0.679~0.836	100.71	0.654	0.766	0.000*
ALB (g/L)	0.716	0.622~0.810	26.75	0.577	0.851	0.000*
CRP/ALB	0.768	0.684~0.850	2.48	0.731	0.649	0.000*
NLR	0.668	0.577~0.759	16.83	0.692	0.649	0.001*
PCT (ng/ml)	0.767	0.684~0.850	0.59	0.673	0.809	0.000*
NT-proBNP (ng/ml)	0.723	0.637~0.809	14265.25	0.635	0.723	0.000*
CRP/ALB+NT-proBNP	0.821	0.751~0.892	-	0.750	0.830	0.000*
CRP/ALB+NLR	0.782	0.707~0.856	-	0.500	0.915	0.000*
NLR+ NT-proBNP	0.741	0.658~0.825	-	0.712	0.670	0.000*
CRP/ALB+NLR+ NT-proBNP	0.830	0.761~0.899	-	0.808	0.777	0.000*

Abbreviations: CRP, C-reaction protein; ALB, Albumin; CRP/ALB, C-reaction protein/Albumin; NLR, Neutrophil-lymphocyte ratio; PCT, procalcitonin; NT-proBNP, N terminal pro B-type natriuretic peptide; PLR, Platelet-lymphocyte ratio; AUC, area under the curve; CI, Confidence interval. *: *P< .*05.

**Table 5:** Prognostic value of NT-proBNP

Optimal cut-off of NT-proBNP, ng/ml	Overall (n=146)	Non-survivors (n=52)	Survivors (n=94)
>14265.25	59 (40.4)	33 (55.9)	26 (44.1)
<14265.25	87 (59.6)	19 (21.8)	68 (78.2)

Abbreviations: NT-proBNP, N terminal pro B-type natriuretic peptide.

## Abbreviations

COPD: Chronic Obstructive Pulmonary Disease
AECOPD: Acute exacerbation of COPD
HF: heart failure
NT-proBNP: N-terminal pro-brain natriuretic peptide
CRP: C-reaction protein
ALB: albumin
NLR: neutrophil-lymphocyte ratio
PLR: platelet-lymphocyte ratio
ICD-10: International Classification of Diseases, Tenth Revision
GOLD: Global Initiative for Chronic Obstructive Lung Disease
PTE: pulmonary thromboembolism
LVEF: left ventricular ejection fraction
CAT: COPD assessment test
LOS: length of stay
NYHA: New York Heart Association
SPSS: Statistical Package for Social Sciences
IQR: interquartile range
ROC: Receiver operating characteristic
mMRC: modified Medical Research Council dyspnea scale
